# Rare Variants in Genes Linked to Appetite Control and Hypothalamic Development in Early-Onset Severe Obesity

**DOI:** 10.3389/fendo.2020.00081

**Published:** 2020-02-21

**Authors:** Petra Loid, Taina Mustila, Riikka E. Mäkitie, Heli Viljakainen, Anders Kämpe, Päivi Tossavainen, Marita Lipsanen-Nyman, Minna Pekkinen, Outi Mäkitie

**Affiliations:** ^1^Children's Hospital, Helsinki University Hospital, Helsinki, Finland; ^2^Folkhälsan Research Center, Genetics Research Program, Helsinki, Finland; ^3^Research Program for Clinical and Molecular Metabolism, Faculty of Medicine, University of Helsinki, Helsinki, Finland; ^4^Department of Pediatrics, Seinäjoki Central Hospital, Seinäjoki, Finland; ^5^City of Turku, Welfare Division, Preventive Healthcare, Turku, Finland; ^6^Molecular Endocrinology Laboratory, Department of Medicine, Hammersmith Campus, Imperial College London, London, United Kingdom; ^7^The Department of Food and Nutrition, University of Helsinki, Helsinki, Finland; ^8^Department of Molecular Medicine and Surgery, Karolinska Institutet, and Department of Clinical Genetics, Karolinska University Hospital, Stockholm, Sweden; ^9^Department of Children and Adolescents, PEDEGO Research Unit, University of Oulu, Oulu, Finland

**Keywords:** childhood obesity, hypothalamus, appetite regulation, hyperphagia, *MC4R*

## Abstract

**Context:** The hypothalamic circuit has an essential role in the regulation of appetite and energy expenditure. Pathogenic variants in genes involved in the hypothalamic leptin–melanocortin pathway, including melanocortin-4-receptor (*MC4R*), have been associated with monogenic obesity.

**Objective:** To determine the rate and spectrum of rare variants in genes involved in melanocortin pathway or hypothalamic development in patients with severe early-onset obesity (height-adjusted weight >60% before age 10 years).

**Methods:** We used a custom-made targeted exome sequencing panel to assess peripheral blood DNA samples for rare (minor allele frequency <0.5%), pathogenic/likely pathogenic variants in 24 genes related to the hypothalamic circuit in 92 subjects (51% males, median age 13.7 years) with early-onset severe obesity (median body mass index (BMI) Z-score + 4.0).

**Results:** We identified a novel frameshift deletion in *MC4R* (p.V103Afs5^*^) in two unrelated patients and a previously reported *MC4R* variant (p.T112M) in one patient. In addition, we identified rare heterozygous missense variants in *ADCY3* (p.G1110R), *MYT1L* (p.R807Q), *ISL1* (p.I347F), *LRP2* (p.R2479I, and p.N3315S) and a hemizygous missense variant in *GRPR* (p.L87M) (each in one patient), possibly contributing to the obesity phenotype in these patients. Altogether 8 % (7/92) of the subjects had rare pathogenic/likely pathogenic variants in the studied genes.

**Conclusions:** Rare genetic variants within the hypothalamic circuit are prevalent and contribute to the development of severe early-onset obesity. Targeted exome sequencing is useful in identifying affected subjects. Further studies are needed to evaluate the variants' clinical significance and to define optimal treatment.

## Introduction

Obesity is a complex disease with various contributing environmental and genetic factors. To uncover the principal genetic factors behind obesity, several advanced research strategies have been used, including twin studies, genome-wide association studies (GWASs), chromosomal microarray analyses and next generation sequencing (1). For example, common single nucleotide polymorphisms (SNPs) in multiple genes have been shown to modulate the risk of obesity, although each of these have only a minor effect on body mass index (BMI) variation (2, 3). We and others have also shown that rare copy number variants (CNVs) are enriched in patients with early-onset severe obesity (4–6). Recently, it has also been suggested that obesity could be a consequence of rare genetic variants with strong effect (7, 8). Despite these advancements, the genetic causes and underlying molecular mechanisms behind obesity are still inadequately understood.

Mutations in genes linked to the leptin-melanocortin signaling in the hypothalamus are associated with monogenic obesity. These genes include i.e., *LEP, LEPR, POMC, PCSK1, MC4R, MC3R, SH2B1, NTRK2, MRAP2*, and *TUB* (1). The discovery of these monogenic mutations has increased our understanding of the mechanisms controlling energy balance. Single-gene mutations are estimated to account for ~7% of cases of non-syndromic severe obesity (1). Of them, *MC4R* mutations are the most frequent with over 300 reported variants (9). The prevalence of pathogenic variants in *MC4R* in Finnish patients with early-onset obesity was reported to be 1.8% (10), and in other populations 1.5–5.8% (7, 11–14). Patients with loss-of-function *MC4R* mutations typically present with severe childhood obesity, hyperphagia, hyperinsulinemia, and increased linear growth (11, 15). MC4R is a G-protein-coupled receptor (GPCR), which activates the stimulatory G protein (Gs), increases cAMP production and activates protein kinase A (PKA), leading to decreased food intake and increased energy expenditure (16). Lately, it has been suggested that in addition to Gs activation the MC4R downstream signaling includes several other pathways potentially important for weight regulation and that more patients than previously assumed may have functionally relevant genetic variants in *MC4R* (16, 17).

SIM1 and BDNF are transcription and neurotropic factors that control the neuronal differentiation of the hypothalamus and mutations in genes encoding these proteins have been associated with obesity (1). Recently, new obesity related genes involved in hypothalamic development or melanocortin pathway have been identified, such as *ADCY3, MYT1L, POU3F2, GRPR*, and *LRP2* (1, 5, 18–22).

In recent years, advances have been made in the development of targeted therapy for patients with rare genetic disorders. Setmelanotide, a MC4R agonist, has shown promising results in promoting weight loss in patients with genetic loss-of-function defects in *POMC, LEPR*, and *MC4R* (9, 23, 24). Genetic testing in patients with severe obesity is therefore important for the patients and their families, as it allows early detection of those at increased risk, optimal targeting of preventive measures and helps to identify patients likely to benefit from pharmacological treatment.

This study evaluated the prevalence and nature of genetic variants within the melanocortin pathway in patients affected by severe early-onset obesity. We have chosen to focus on the most severe end of the spectrum of obesity, namely on forms presenting already in early childhood, as they are more likely to be explained by genetic causes. We evaluated a Finnish cohort of 92 patients with severe childhood obesity and aimed to determine the rate and spectrum of rare pathogenic/likely pathogenic genetic variants in 24 genes linked to the melanocortin pathway or hypothalamic development.

## Materials and Methods

### Study Subjects

This study included altogether 92 children and adolescents with severe early-onset childhood obesity, as defined according to the Finnish growth standards (25) as height-adjusted weight >60% before 10 years of age. All children were born to non-consanguineous parents. The patients were recruited at Children's Hospital at Helsinki University Hospital, Seinäjoki Central Hospital and Department of Children and Adolescents at Oulu University Hospital in Finland during years 2011–2017. All participating patients had been followed up by a pediatrician and patients diagnosed with an underlying endocrine or genetic disorder were excluded from the study (e.g., Prader–Willi syndrome, hypercortisolism, and hypothyroidism). Ethical approvals for the study were obtained from the research ethics committees of the Hospital District of Helsinki and Uusimaa, the Pirkanmaa Hospital District and the Northern Ostrobothnia Hospital District. Informed written consents were obtained from all the participants or from one of their guardians (for subjects aged <18 years).

Hospital records were reviewed for clinical data. Anthropometric measurements including height, weight and waist circumference were measured during a study visit. BMI was calculated as weight divided by square height (kg/m^2^). Sex-and age-specific BMI Z-scores were derived based on the World Health Organization reference values (www.who.int/childgrowth/standards). Blood samples were collected for genetic studies from all participants and from available first-degree family members (parents and siblings). Heights and weights were collected from the participating family members.

### Genes in the Panel

**Figure 1:**
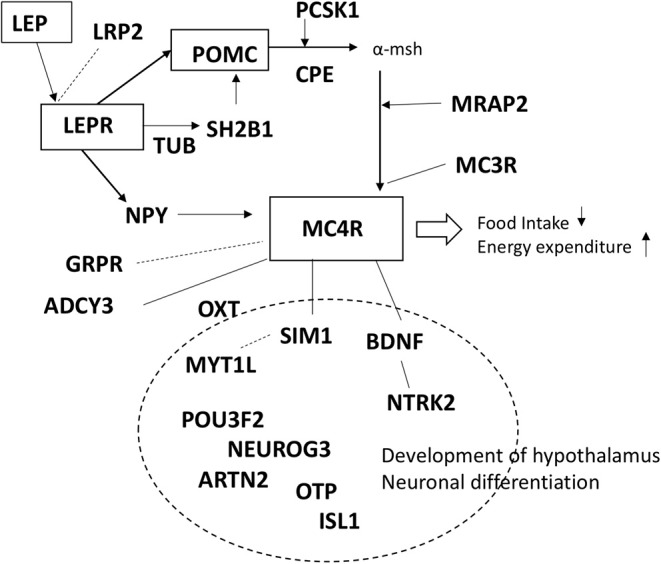
Schematic presentation of the genes included in the panel.

We performed a literature search to identify genes related to the melanocortin pathway and the development of the hypothalamus (1, 5, 12, 18, 19, 21, 26–33). The 24 genes included in the panel were: (1) genes in which variants have previously been reported to cause or associate with obesity (*ADCY3, BDNF, CPE, GRPR, LEP, LEPR, LRP2, MC3R, MC4R, MRAP2, MYT1L, NPY, NTRK2, PCSK1, POMC, SH2B1, SIM1, TUB*) and (2) genes previously reported in animal models/linkage analysis/CNV studies to be involved in the melanocortin pathway or development of hypothalamus (*ARNT2, ISL1, NEUROG3, OTP, OXT, POU3F2*). Summary of the genes in the panel are presented in Table 1 and Figure 1.

**Table 1:** List of the obesity related genes included in the panel.

**Gene symbol**	**Gene name**	**Function related to appetite control or hypothalamic development**	**Relevance for human obesity**
*ADCY3*	Adenylate cyclase 3	Involved in Gs signaling in hypothalamus, catalyzes the synthesis of cAMP	Variants associated with obesity
*ARNT2*	Aryl hydrocarbon receptor nuclear translocator 2	Regulates neuronal differentiation of hypothalamus	Role in the development of hypothalamus, animal models
*BDNF*	Brain-derived neurotrophic factor	Regulates neuronal differentiation of hypothalamus	Disease causing
*CPE*	Carboxypeptidase E	Peptide processing enzyme, cleaves neuropeptides	Variants associated with obesity
*GRPR*	Gastrin releasing peptide receptor	Involved in the regulation of satiety	Variants associated with obesity
*ISL1*	ISL LIM homeobox 1	Involved in differentiation of hypothalamic neurons and expression of *POMC*	Role in the development of hypothalamus, linkage analysis
*LEP*	Leptin	Inhibits food intake	Disease causing
*LEPR*	Leptin receptor	Leptin receptor	Disease causing
*LRP2*	LDL receptor related protein 2	Increases leptin-induced STAT3 activation in POMC-expressing neurons	Variants associated with obesity
*MC3R*	Melanocortin-3 receptor	Regulates energy homeostasis	Variants associated with obesity
*MC4R*	Melanocortin-4 receptor	Regulates appetite and energy homeostasis	Disease causing
*MRAP2*	Melanocortin 2 receptor accessory protein	Regulates the function of melanocortin receptors	Disease causing
*MYT1L*	Myelin transcription factor 1 like	Transcription factor involved in development of hypothalamus	Variants associated with obesity
*NEUROG3*	Neurogenin 3	Transcription factor involved in development of hypothalamus	Role in the development of hypothalamus, animal models
*NPY*	Neuropeptide Y	Stimulates food intake	Variants associated with obesity
*NTRK2*	Neurotrophic tyrosine kinase receptor type 2	Regulates neuronal differentiation of hypothalamus, BDNF receptor	Disease causing
*OTP*	Orthopedia homeobox	Transcription factor involved in development of hypothalamus	Role in the development of hypothalamus, animal models
*OXT*	Oxytocin	Involved in appetite regulation	Role in hypothalamic circuit, animal models
*PCSK1*	Proprotein convertase subtilisin/kexin type 1	Involved in activation of processing of proteins and neuropeptide precursors	Disease causing
*POMC*	Pro-opiomelanocortin	Protein precursor cleaved into alfa-MSH and binds to MC4R	Disease causing
*POU3F2*	POU class 3 homeobox 2	Transcription factor involved in development of hypothalamus	Role in development of hypothalamus, CNV studies
*SH2B1*	Src homology 2 B adapter protein 1	Involved in modulation of leptin signaling	Disease causing
*SIM1*	Single-minded homolog 1	Regulates neuronal differentiation of hypothalamus	Disease causing
*TUB*	Tubby bipartite transcription factor	Involved in regulation of neuropeptides	Variants associated with obesity

### Genetic Analysis

Genomic DNA was isolated from obtained peripheral blood samples according to standard procedures. The probes for targeted exome sequencing were designed using SeqCap EZ Choice Library and NimbleDesign (Roche NimbleGen, United States). DNA capture and Next generation sequencing were performed at Oxford Genomics Center. Sequence reads were aligned to the reference human genome GRCh37. Variants were called using software Platypus software (version 0.8.1) and functionally annotated using Ensembl Variant Effector Predictor (VEP). Variant filtering was performed using VarAFT (version 2.13) (34). Non-synonymous exonic and splice site variants were considered. Variants with allele frequency higher than 0.5% (in Exome Aggregation Consortium (http://exac.broadinstitute.org), the Genome Aggregation Database (http://gnomad.broadinstitute.org), 1,000 Genomes Project (http://www.internationalgenome.org) and the Sequencing Initiative Suomi project (SISu) (http://sisuproject.fi) were filtered out. Possible pathogenicity of the variants was assessed using different variant prediction databases (Polyphen-2, SIFT, Combined Annotation Dependent Depletion (CADD) and MutationTaster2). We considered rare variants with allele frequency <0.5% and variants predicted to be pathogenic by several *in silico* prediction tools, including a CADD score >20, as likely pathogenic.

Sanger sequencing was performed to confirm the findings and to analyze parental and siblings' samples to determine inheritance pattern. Primers were designed using Primer3 software and PCR was performed using DreamTaq^TM^ DNA Polymerase (Thermo Fischer Scientific) according to standard protocol. Chromatograms were analyzed with Sequencer v5.0 software. Primer sequences are available upon request.

### Statistical Analyses

Results are reported as medians (interquartile range, IQR). Statistical analyses were performed with SPSS Statistical package (version 25.0.0.1).

## Results

### Characteristics of the Study Cohort

The study included a total of 92 subjects (51% males). At the time of the study visit their median age was 13.7 years (IQR 10.6–16.8 years) and median BMI Z-score +4.0 (IQR + 3.4 to + 4.9). The study subjects fulfilled our inclusion criteria (height-adjusted weight >60 %) at the median age of 6 years (IQR 4.5–7.0 years).

### Targeted Exome Sequencing

On average 99.3% of bases in the coding exons were covered by at least 100 reads and the mean read depth of coverage was 755X. We found rare pathogenic/likely pathogenic variants in 7/92 (8%) of the study subjects and one rare variant of uncertain significance in one patient. The rare variants were in six of the 24 genes in the panel, with variants in the *MC4R* gene being the most common finding (3/92). The genetic and clinical features of the patients with the rare variants are presented below and summarized in Table 2. The prediction values of the identified variants are presented in Table 3.

**Table 2:** Genetic and clinical characteristics of the patients with rare genetic variants (MAF < 0.5%), reference human genome GRCh37.

**Patient**	**Gene**	**Type**	**Chromosome position**	**Transcript**	**cDNA**	**Protein**	**Inheritance**	**BMI (*Z*-score)**	**Clinical features**
1	*MC4R*	Frameshift deletion	18:58039275 A>-	NM_005912	308delT	V103Afs5*	Maternal	4.3	Hyperphagia, type 2 diabetes
2	*MC4R*	Frameshift deletion	18:58039275 A>-	NM_005912	308delT	V103Afs5*	*De novo*	3.0	Hyperphagia
3	*MC4R*	Missense variant	18:58039248 G>A	NM_005912	335C>T	T112M	NA	4.1	
4	*ADCY3*	Missense variant	2:25042908 C>G	NM_004036	3328G>C	G1110R	Maternal	4.8	Insulin resistance, asthma, depression
5	*MYT1L*	Missense variant	2:1893113 C>T	NM_001303052	2420G>A	R807Q	NA	3.8	Behavioral problems
6	*ISL1*	Missense variant	5:50689433 A>T	NM_002202	1039A>T	I347F	NA	2.8	
7	*LRP2*	Missense variant	2:170062653 C>A	NM_004525	7436G>T	R2479I	Maternal	2.8	
8	*LRP2*	Missense variant	2:170038731 T>C	NM_004525	9944A>G	N3315S	NA	4.3	Insulin resistance, hypothyroidism, depression
8	*GRPR*	Missense variant	X:16142335C>A	NM_005314	259C>A	L87M	NA	4.3	Insulin resistance, hypothyroidism, depression

*NA, data not available*.

**Table 3:** Prediction values of the rare genetic variants detected in the obesity cohort.

**Gene**	**Variant**	**Polyphen-2**	**SIFT**	**CADD**	**MutationTaster**
*MC4R*	V103Afs5*	Not applicable	Not applicable	Not applicable	Disease causing
*MC4R*	T112M	Possibly damaging	Tolerate	8.9	Polymorphism
*ADCY3*	G1110R	Probably damaging	Deleterious	34.0	Disease causing
*MYT1L*	R807Q	Possibly damaging	Tolerate	23.3	Disease causing
*ISL1*	I347F	Possibly damaging	Deleterious	23.0	Disease causing
*LRP2*	R2479I	Probably damaging	Deleterious	34.0	Disease causing
*LRP2*	N3315S	Probably damaging	Deleterious	25.6	Disease causing
*GRPR*	L87M	Probably damaging	Deleterious	25.6	Disease causing

## Rare Exonic Variants and Associated Phenotypes

### *MC4R* Variants

**Figure 2:**
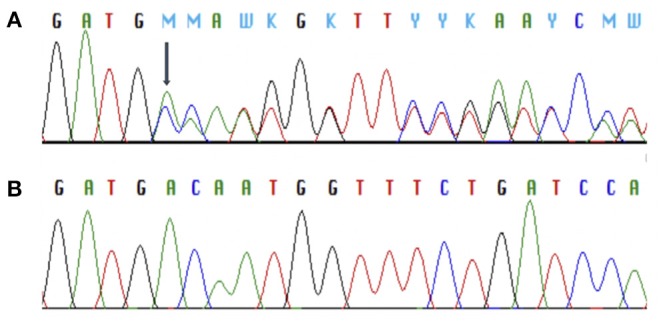
Chromatograms of Sanger sequence analysis of *MC4R* showing a heterozygous frameshift deletion (p.V103Afs5*) in two index patients with monogenic obesity **(A)** and the normal MC4R sequence **(B)**.

**Figure 3:**
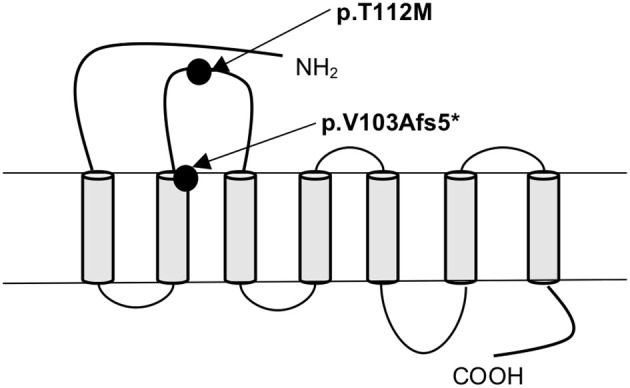
Schematic presentation of the MC4R protein and the location of the frameshift variant (p.V103Afs5*) and the missense variant (p.T112M) identified in patients with obesity.

In *MC4R* (NM_005912) we identified altogether 2 variants. The first was a novel heterozygous frameshift deletion c.308delT present in two unrelated patients. The frameshift variant was confirmed by Sanger sequencing (Figure 2). The deletion causes a frameshift at amino acid 103 (p.V103Afs5^*^) and introduces four new amino acids resulting in a stop codon in the region of the second transmembrane domain, leading to a significantly truncated protein of only 107 amino acids compared to the normal 332-amino-acid MC4R (Figure 3). The variant is absent in all databases (ExAC, gnomAD, 1,000 Genomes, SISu). MutationTaster2 predicted the deletion as disease-causing.

**Figure 4:**
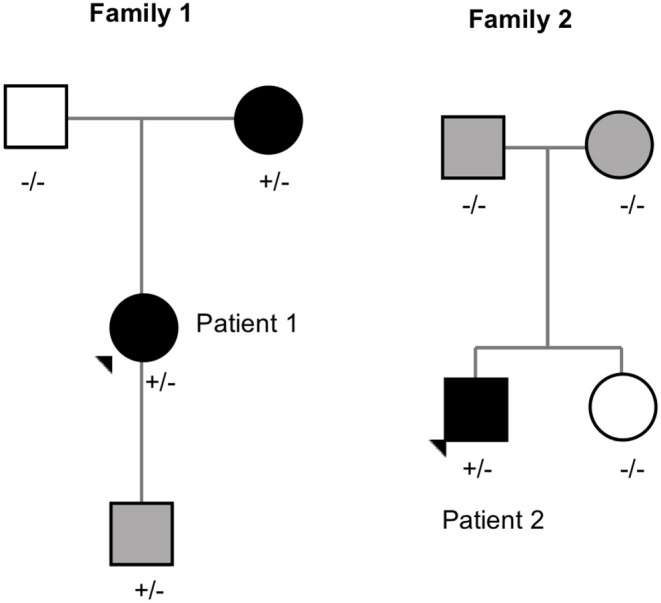
Pedigrees of the families with MC4R frameshift variant (p.V103Afs5*). Square = male, circle = female, +/– heterozygote carrier, –/– wildtype. Black square/circle = obesity, gray square/circle = overweight, white square/circle = normal weight.

The pedigrees of the two unrelated families with the *MC4R* frameshift variant are presented in Figure 4.

Patient 1 is a 20-year-old female with normal birth measurements: weight 3.6 kg and length 48 cm. She presented with severe obesity already at the age of 1 year with weight 17.8 kg and height 81.5 cm (BMI Z-score + 5.8). Presently at age 20 years, her weight is 121 kg, height 170.5 cm (BMI 44) and waist circumference 133 cm. She also has type 2 diabetes and hyperphagia. Her mother, heterozygous for the same mutation, presented with obesity in childhood and overweight during adulthood. The mutation-negative father had normal weight during childhood. The index patient's 2.5-year-old son also carries the same frameshift deletion. He presented with overweight at age 9 months with a BMI Z-score of + 2.9. Currently, his BMI Z-score is +1.8 and he requires close monitoring for pronounced hyperphagia.

Patient 2 is an 18-year-old male with BMI 35 (weight 106 kg and height 173.5 cm) and waist circumference 110 cm. His birth measurements were normal (3.4 kg and 50.5 cm) and he presented with obesity at the age of 3 years measuring 18.5 kg and 93.5 cm (BMI Z-score +3.9). He also presented with hyperphagia. The variant was *de novo* as his overweight parents and normal weight sister did not have the variant.

The other identified *MC4R* variant is a previously reported heterozygous missense variant, c.335C>T (p.T112M) (Figure 3) in Patient 3; a 12-year-old boy with normal birth measurements (3.5 kg and 50 cm). He presented with obesity at age 4 years measuring 29.2 kg and 114.3 cm (BMI Z-score +4.5). Presently at the age of 12 years, his weight is 114.4 kg and height 177.1 cm (BMI Z-score +4.1). He had accelerated linear growth with growth velocity of 12 cm/year at the age of 5 years, leading to height-for-age Z-score +4.1 at age 12 years. His mother's height was 163 cm and father's 183 cm. The patient's mother with obesity did not harbor the variant. No DNA was available from the other family members. The allele frequency of the variant is 0.09% in all populations in gnomAD, 0.2% in the Finnish population in gnomAD and 0.18% in SISu.

### *ADCY3* Missense Variant

We identified a heterozygous missense variant in *ADCY3* (c.3328G>C, p.G1110R) in Patient 4; a 16-year-old female with a BMI Z-score of +4.8, insulin resistance, asthma and depression. She had normal birth measurements (3.25 kg and 51 cm). She presented with obesity already at the age of 1.5 years measuring 16.3 kg and 86.4 cm (BMI Z-score + 3.6). The patient's mother (BMI 35) also carried the same variant. The father's DNA was not available. According to gnomAD, the variant is found in 1/3474 in the Finnish population.

### *MYT1L* Missense Variant

We detected a heterozygous missense variant in *MYT1L* (c.2420G>A, p.R807Q) in Patient 5; a 12-year-old girl with a BMI Z-score of + 3.8. She presented with aggressive behavior and reading problems. The variant was not found in her normal-weight mother; DNA of the normal-weight father was not available. The allele frequency of the variant is 0.01% in all populations in gnomAD, 0.1% in the Finnish population in gnomAD and 0.1% in SISu.

### *ISL1* Missense Variant

We found a heterozygous missense variant in *ISL1* (c.1039A>T, p.I347F) in Patient 6; a 9-year-old girl with a BMI Z-score of + 2.8. The patient had normal development and no endocrinological abnormalities. Her mother (BMI 39) was also a carrier of the variant but her normal-weight father was not. The variant is found in 5/25038 (all heterozygotes) in the Finnish population in gnomAD and the allele frequency is 0.03 % in SISu.

### *LRP2* Missense Variants

We identified two heterozygous missense variants in *LRP2*. The first variant (c.7436G>T, p.R2479I) was found in Patient 7, a 19-year-old boy with a BMI Z-score of + 2.8, and in his mother (BMI 39). The variant is not found in any of the above-mentioned databases.

The second variant in *LRP2* (c.9944A>G, p.N3315S) was detected in Patient 8; a 16-year-old boy with a BMI Z-score of + 4.3, insulin resistance, fatty liver, hypothyroidism and severe depression. The patient had an overweight mother and a normal-weight father, but parental DNAs were unavailable. The variant is not found in the Finnish population in gnomAD or in SISu, but it is found in 2/24956 (both heterozygotes) in African population in gnomAD.

### *GRPR* Missense Variant

In Patient 8, harboring an *LRP2* variant (p.N3315S), we also identified a hemizygous missense variant (c.259C>A, p.L87M) in *GRPR*. This variant is present with allele frequency 0.02% in the Finnish population in gnomAD and SISu.

## Discussion

Recent advances in genetic methodology have enabled identification of novel genetic factors that may contribute to obesity. Such discoveries have increased our understanding of the significance of various components involved in the hypothalamic circuit regulating appetite and energy expenditure. By using targeted exome sequencing, we identified several rare exonic variants in genes related to the leptin-melanocortin pathway and hypothalamic development in a large cohort of Finnish patients with severe childhood-onset obesity. Altogether 7 of the 92 studied subjects (8%) harbored at least one rare and pathogenic/likely pathogenic variant in the studied 24 genes, suggesting that a significant proportion of severe childhood-onset obesity could be related to genetic defects within the hypothalamic circuit.

The prevalence of monogenic obesity varies with ethnicity and consanguinity. Close consanguinity is rare in the Finnish population (35) and there were no consanguineous families in our cohort. We did not detect any pathogenic variants in LEPR, LEP, POMC, and PCSK1 genes. Patients with these gene defects often have very early and rapid weight gain and associated endocrinopathies. It is therefore possible, that such patients may have been identified earlier by pediatricians and thus excluded from our study focusing on obesity of unknown etiology.

The prevalence of pathogenic variants in *MC4R* in our cohort was 2% (2/92), which is comparable to previous reports (7, 10–14). The patients in our study with the novel *MC4R* frameshift deletion had severe childhood obesity and hyperphagia. The frameshift deletion is considered pathogenic as it leads to a significantly truncated protein and its functional consequences on the receptor function are likely to be considerable.

Additionally, we detected a previously identified rare heterozygous missense variant (p.T112M) in *MC4R*. Our patient presented with childhood obesity, markedly increased linear growth and tall stature during childhood. *MC4R* deficiency has been associated with accelerated linear growth and increased final height (15). In contrast, we did not observe increased final height or linear growth in the patients with the *MC4R* frameshift variant. The missense variant is not shown to be pathogenic according to prediction tools and the variant has previously been reported as having normal Gs signaling according to *in vitro* functional studies (10, 36). However, the variant has been described as a biased signaling variant that leads to decreased ERK1/2 activation. *MC4R* variants that alter the ERK1/2 signaling have been suggested to be involved in regulation of food intake (16, 17). We considered this variant to be of uncertain significance as further studies are needed to explore the impact of non-Gs signaling *MC4R* variants on weight regulation.

*ADCY3* encodes adenylyl cyclase 3, which localizes at the cilia of paraventricular nucleus in the hypothalamus and is involved in the Gs signaling. Previous studies have shown that *Adcy3* heterozygous null mice present with obesity, insulin resistance and increased adiposity (37). A glucagon-like peptide-1 analog, liraglutide, has been shown to increase the expression of hepatic *Adcy3* in obese and diabetic mice and the *Adcy3* activation may partly account for the reduction of blood glucose levels and bodyweight (38). Furthermore, previous studies have identified homozygous mutations in *ADCY3* in children with severe obesity (33), and an *ADCY3* variant has been associated with increased risk of obesity and type 2 diabetes in homozygous individuals and to a lesser degree in heterozygous carriers (18). The patient in our study was heterozygous for a non-synonymous *ADCY3* variant with a high CADD score. She had severe obesity and insulin resistance and her mother, harboring the same variant, also presented with obesity. The *ADCY3* variant was predicted disease causing and is likely to play a role in the obesity and insulin resistance in our patient.

*MYT1L* is expressed in the brain and involved in the development of neuroendocrine hypothalamus. Several *MYT1L* mutations have been identified in patients with obesity, intellectual disability, developmental delay, autistic features and behavioral problems (19, 20, 39). Loss of *MYT1L* in zebrafish was related to altered hypothalamic development and loss of oxytocin (*OXT*) in the neuroendocrine preoptic area. *MYT1L* was demonstrated to be part of the melanocortin pathway, functioning downstream of *SIM1*, and *OXT* was recognized as a potential treatment target (19). We have previously described a Finnish patient with a frameshift deletion in *MYT1L* presenting with obesity, intellectual disability and developmental delay (20). The patient in this study with the presumed *de novo MYT1L* variant had severe obesity, aggressive behavior and learning difficulties. Although our patient did not present with marked developmental delay or intellectual disability, her aggressive behavior, learning difficulties and the early-onset obesity are likely to be related to the identified *MYT1L* variant.

The Insulin gene enhance protein ISL-1, encoded by *ISL1*, is a transcription factor involved in many developmental processes. Mice and zebrafish experiments have shown that *Isl1* is expressed in the developing brain, regulates the differentiation of hypothalamic neurons and is important for the expression of *Pomc* in the hypothalamus. Inactivation of *Isl1* in Pomc neurons reduces the expression of *Pomc* and leads to obesity (32, 40). A linkage analysis in French families with morbid obesity showed linkage between *ISL1* and BMI and leptin (41). Variants in *ISL1* have previously been associated with congenital heart defects (42), but to our knowledge, no mutations in *ISL1* have been reported in monogenic obesity. The *ISL1* missense variant found in our study segregated with the obesity phenotype in the family and was predicted disease causing. The role of *ISL1* in the development of obesity and the functional relevance of the variant require further studies.

Low-density lipoprotein receptor 2 gene (*LRP2*), encodes an endocytic receptor, LRP2 or megalin. LRP2 has many ligands in various tissues. LRP2 increases leptin-induced STAT3 activation in POMC-expressing neurons in hypothalamus and inhibits food intake. STAT3 signaling is reduced in the absence of functional LRP2 (22). *LRP2* has been associated with monogenic obesity (1, 22). Here we identified two patients with rare heterozygous variants in *LRP2*; one novel missense variant (p.R2479I), and another rare missense variant (p.N3315S), neither one has previously been found in the Finnish population. Both *LRP2* variants were predicted disease-causing. More studies are required to explore the functional significance of these *LRP2* variants.

*GRPR*, encoding gastrin-releasing peptide receptor, is an X-linked 7-transmembrane G-protein coupled receptor that activates the phospholipase C signaling pathway. *GRPR* is expressed in several tissues, including the brain and is involved in the regulation of satiety. A previous study identified in five patients with obesity heterozygous or hemizygous mutations in *GRPR* that were predicted functionally relevant (5). The hemizygous *GRPR* variant (p.L87M) identified in our study was also detected in a boy with severe obesity by Serra-Juhe et al. (5). In their co-expression analysis *GRPR* showed clear enrichment of co-expression with genes from the melanocortin pathway (5). Our patient presented with early-onset obesity, insulin resistance, fatty liver, hypothyroidism, and severe depression. Interestingly, *GRPR* has also been implicated in psychiatric disorders like depression (43), suggesting that the hemizygous *GRPR* variant contributes to the severe depression seen in our patient. In addition, our patient also harbors a rare variant (p.N3315S) in the *LRP2* gene and it therefore remains uncertain how each of the two variants contribute to the phenotype.

Next generation sequencing is an important tool in identifying genetic causes in monogenic obesity. Targeted exome sequencing provides several advantages compared to whole-exome sequencing; it is cost-effective, has high quality with high read depth and coverage, data interpretation is easier as the number of genes is limited, and chance of incidental findings is minimal. Its weakness is the limited number of genes included in panel. Because of this methodological limitation we may have missed variants in novel genes that were not included in the gene panel but still contribute to hypothalamic function. Another limitation in our study is the lack of available family members for genetic testing in some instances and therefore interpretation of the pathogenicity of the identified variants was more challenging. Further, our study did not include any functional evaluations of the genetic findings and therefore the contribution of some of the variants to the obesity development remains uncertain. However, we only considered rare exonic variants with allele frequency <0.5% and variants predicted to be pathogenic by several *in silico* prediction tools, including a high CADD score as likely pathogenic.

In conclusion, we report rare pathogenic/likely pathogenic variants in genes involved in hypothalamic circuit in 7/92 (8%) of patients with early-onset obesity. Our results support the role of hypothalamic melanocortin pathway in the development of obesity. Further studies are required to explore the clinical relevance of the rare genetic variants. Novel therapies targeting specific genetic defects are under development and may offer future treatment modalities in patients with monogenic obesity.

## Data Availability Statement

Data cannot be shared publicly because the data consists of sensitive patient data. More specifically the data consists of individual clinical data and individual genotypes for young children. Data are available from the Helsinki University Hospital's Institutional Data Access/Ethics Committee for researchers who meet the criteria for access to confidential data. Data availability contacts: Outi Mäkitie, outi.makitie@helsinki.fi.

## Author Contributions

PL, TM, HV, ML-N, MP, and OM: study design. PL, TM, HV, PT, and ML-N: data collection. PL, AK, MP, and OM: data analysis and data interpretation. PL, RM, AK, MP, and OM: drafting of manuscript. All authors contributed to manuscript revision and approved the final version.

### Conflict of Interest

OM declares consultancy to Kyowa Kirin, Alexion, and Sandoz. The remaining authors declare that the research was conducted in the absence of any commercial or financial relationships that could be construed as a potential conflict of interest.
